# DELTO Study: Delphi Consensus on Long-Term Textbook Outcome After Metabolic Bariatric Surgery

**DOI:** 10.1007/s11695-024-07587-6

**Published:** 2025-01-18

**Authors:** Ellen A. M. Kuipers, Lindsy van der Laan, Mirjam A. Kaijser, Josien G. Timmerman, Nic Veeger, André P. van Beek, Marloes Emous, Marc J. van Det

**Affiliations:** 1 Ziekenhuis Groep Twente, Almelo, Netherlands; 2 University of Twente, Enschede, Netherlands; 3 University Medical Center Groningen, Groningen, Netherlands; 4 Medical Center Leeuwarden, Leeuwarden, Netherlands

**Keywords:** Metabolic Bariatric Surgery, Core Outcome Set, Long-term outcome, Quality of life, Weight loss, Remission of comorbidities, Complications, Delphi method

## Abstract

**Background:**

This study aimed to create a comprehensive Core Outcome Set (COS) for assessing the long-term outcome (≥ 5 years) after Metabolic Bariatric Surgery (MBS), through the use of the Delphi method.

**Methods:**

The study utilized a three-phase approach. In Phase 1, a long list of items was identified through a literature review and expert input, forming the basis for an online Delphi survey. In Phase 2, Dutch healthcare professionals involved in MBS care, defined as having at least 1 year of experience in routine follow-up or managing issues arising during follow-up, rated the importance of these items over three Delphi rounds using a 5-point Likert scale. Participants had the option to suggest additional items. Consensus was defined as 75% agreement among panelists. In Phase 3, the final COS was validated at a national conference.

**Results:**

Thirty-one professionals participated in the first Delphi round. Of these, 28 (90%) completed the second round, and 24 (77%) completed the third round. The final COS, validated by 18 healthcare professionals, included various domains: short-term textbook outcome, weight loss, remission of comorbidities, quality of life, micronutrient deficiencies, lifestyle, psychopathology, long-term complications, and preoperative indication.

**Conclusions:**

The final COS offers a multidimensional approach to evaluate long-term outcomes after MBS. This COS is expected to enhance the measurement and benchmarking of MBS care, providing a more holistic view of patient outcomes.

**Supplementary Information:**

The online version contains supplementary material available at 10.1007/s11695-024-07587-6.

## Introduction

Metabolic Bariatric Surgery (MBS) has proven its long-term efficacy as a treatment option for patients with severe obesity, addressing both weight loss and the improvement of obesity-related comorbidities [1]. Current literature predominantly focuses on weight loss as the primary outcome, with certain papers providing data on the evolution of associated comorbidities [1–3]. Nevertheless, it is imperative to acknowledge that MBS is an invasive intervention influencing various aspects of a patient’s life, including mental health, dietary patterns, and overall quality of life (QoL) [2, 4]. This indicates that there are other valued outcome measures beyond weight loss and comorbidity reduction, that contribute to the long-term success of MBS [5–7]. Therefore, a more holistic assessment of outcomes, encompassing physical, mental, and quality of life aspects, is crucial to evaluate the long-term success of MBS comprehensively.

Textbook Outcome (TO) serves as a comprehensive outcome indicator, encapsulating the most favorable outcome for a patient within a single measure [8]. TO was initially introduced in 2013 in colorectal surgery by Kolfschoten et al. [8] to evaluate variations among hospitals in achieving TO after colon surgery for malignancies and to make hospital comparison achievable [8]. Over the years, TO has been adopted across various surgical fields, including esophagogastric cancer [9], elective aneurysm repair [10], and non-small-cell lung cancer [11]. Since TO mirrors an optimal postoperative course, encompassing diverse process parameters, it proves valuable for monitoring the quality of care and consequently driving quality improvement.

In MBS, Poelemeijer et al. [12] introduced a TO measure to provide insights into short-term postoperative outcomes, offering a perspective on the quality of care. The majority, if not all, of the TO measures, typically depict an ideal short-term postoperative trajectory (within 30 days after surgery) [8–12]. Currently, there is no TO measure to evaluate the long-term outcome after MBS, incorporating patient-related facets. Furthermore, there is no agreement on the specific parameters to be included in a long-term TO after MBS. We anticipate that developing a TO for the long-term outcome after MBS will be challenging due to the extensive impact MBS has on a patient’s life. As a single measure, TO may not adequately capture the multifaceted outcome. Hence, it is likely more informative and beneficial to develop a multidimensional Core Outcome Set (COS) for evaluating the long-term outcome. A COS is a standardized set of outcomes, agreed upon by stakeholders, that should be measured and reported for a specific condition, ensuring comprehensive and consistent assessment.

In 2016, Coulman et al. [13] developed a COS to evaluate the benefits and adverse events after MBS. Their final COS included nine key outcomes: weight, diabetes status, cardiovascular risk, overall quality of life, mortality, technical complications of the specific operation, any re-operation/re-intervention, dysphagia/regurgitation, and micronutrient status [13]. While this COS provides a solid foundation for use in MBS trials, there remains a need for a clinical tool tailored to everyday practice to assess the long-term outcomes after MBS. This is particularly important as MBS patients are typically young, with many years ahead of them, potentially facing long-term complications. Moreover, lifelong adjustments to lifestyle are required. The ultimate goal of MBS is to improve overall health and enable patients to actively participate in society, rather than focusing solely on weight loss. Therefore, a clinical tool to assess outcomes in the long term should incorporate additional factors, including more relevant obesity-related comorbidities, long-term complications, but also lifestyle factors, to ensure comprehensive patient care and outcome assessment. Most of these factors were assessed in the study by Coulman et al. [14]. However, their focus was not on daily clinical practice, and they did not specifically focus on the long-term outcomes (≥ 5 years) after MBS.

To create a standardized set of parameters to evaluate long-term outcomes after MBS, the Delphi method is a suitable approach. In healthcare contexts, the expert Delphi process is commonly employed to establish consensus on predefined clinical issues [15–17]. This research technique involves an iterative process aimed at achieving consensus among an expert panel, particularly in situations with limited evidence where expert opinion holds significance [18]. The Delphi technique has demonstrated its utility in various surgical healthcare settings for selecting quality indicators, making it a suitable method for developing a COS that seeks input from professionals’ opinions and experiences [9–11].

This study aims to create a comprehensive COS for assessing the long-term outcome (≥ 5 years) after MBS, incorporating patient-related factors through the use of the Delphi method. This COS can add value to clinical practice to compare or improve results after MBS.

## Methods

### Phase 1: Identification of Long List of Outcomes and Development of Survey Questionnaire

Outcomes following MBS were identified from two sources: a literature search via PubMed and the clinical expertise of the authors. Two researchers (E. K. and L. v. d. L.) categorized these outcomes, which were subsequently discussed with the other authors. The finalized categories were then operationalized into questionnaire items for the first Delphi round.

Questionnaires for the subsequent Delphi rounds were based on the findings of the previous Delphi rounds. All questionnaires were administered in Dutch, the participant’s native language. Castor (CDM 2023.4) was used for the construction and distribution of the questionnaires.

### Phase 2: Delphi Process

#### Expert Panel

Dutch healthcare professionals closely involved in the care of patients after MBS were invited to join the expert panel. Invitations were sent to metabolic bariatric surgeons, internists/endocrinologists, Physician Assistants (PAs), and other physicians involved in metabolic bariatric care through their respective national organizations dedicated to obesity and MBS. Inclusion criteria were [1] involvement in routine follow-up or management of complications following MBS, and [2] at least 1 year of experience in follow-up after MBS. Completing the questionnaire implied informed consent, as outlined in the introduction of the questionnaire.

#### Delphi Processes

In each Delphi round, participants rated the importance of each long-term outcome item on a 5-point Likert scale, ranging from “not important at all” to “very important.” Participants could provide additional comments for each domain if deemed necessary. Each round allowed participants 3 weeks to complete the questionnaire, with reminders sent after 7 and 14 days. The consensus was defined as [1] at least 75% of participants rating an item as 1 (“not important at all”) or 2 (“not important”), or [2] at least 75% of participants rating an item as 4 (“important”) or 5 (“very important”) [19, 20]. Items that reached consensus as important or not important were included or excluded from the COS, respectively, and were not presented in subsequent rounds, except during the validation round.

After each round, statistical analysis was performed, and a revised questionnaire was created, presenting the remaining items for reassessment. For multiple-choice questions, the two or three most common answers from the previous round were presented again, along with aggregated group feedback showing the percentage of responses for each item. Participants who did not respond to either the first or second questionnaire were deemed ineligible for subsequent rounds, except for the validation round.

### Phase 3: Validation

The validation round took place at the Dutch Society for Metabolic and Bariatric Surgery (DSMBS) conference in April 2024, which was held in Veenendaal, the Netherlands. We requested attendees to validate our COS. Recruitment involved providing study information and access to the questionnaire via a QR code displayed on a slide or flyer. Interested participants could read the study details. If they chose to participate, completing the questionnaire indicated informed consent, as stated in the introduction of the questionnaire. All items classified as important during any of the Delphi rounds were included in the validation round. Each domain was evaluated with a single question, asking participants to indicate their agreement with the final COS items. One week after the conference, a reminder was sent by email to the conference attendees via the organizing committee to encourage additional participation.

#### Sample Size

There are no established guidelines for determining the sample size for Delphi surveys, nor is there a requirement for a statistically representative sample [21]. Consequently, an opportunistic recruitment strategy was employed. All Dutch healthcare professionals closely involved in the care of patients after MBS were approached, aiming to recruit as many relevant experts as possible.

## Results

### Phase 1: Identification of Long List of Outcomes and Development of Survey Questionnaire

A review of the literature and clinical expertise identified ten domains, which included short-term TO, weight, comorbidities, QoL, micronutrient deficiencies, lifestyle, psychopathology, short- and long-term complications, and readmission (Table 1). For the survey, relevant items were categorized into the identified domains.

**Table 1:** Survey questionnaire items for round 1

Domain	Item
Short-term textbook outcome	1. Prolonged length of stay (> 2 days) 2. Readmission 3. Severe postoperative complications (CD grade III or IV) 4. Re-intervention 5. Intensive care observation 6. Mortality
Weight	1. Weight loss 2. Term to express weight loss 3. Cutoff valuefor a clinical optimal response 4. Recurrent weight gain 5. Term to express recurrent weight gain 6. Cutoff valuefor recurrent weight gain
Comorbidities	Remission or improvement of the comorbidity: 1. Hypertension 2. Dyslipidemia 3. Osteoarthritis 4. GERD 5. OSAS 6. Diabetes mellitus 7. Asthma/COPD
Quality of life	1. Quality of life 2. Questionnaire to assess QoL 3. Method of evaluating QoL
Micronutrient deficiencies	1. Micronutrient deficiencies 2. Severity of the deficiency
Lifestyle	1. Sufficient exercise 2. Sufficient mealtimes per day 3. Sufficient protein intake per day 4. Cutoff value for sufficient protein intake per day 5. Separate eating and drinking 6. Avoidance of carbonated beverages
Psychopathology	1. Deterioration of pre-existing psychopathology 2. Problems with binge eating disorder 3. Severity of binge eating disorder 4. Problems with anorexia nervosa 5. Severity of anorexia nervosa 6. Problems with depression/depressive symptoms 7. Severity of depression/depressive symptoms 8. Development of a new addiction
Short-term complications	1. Hemorrhage 2. Anastomotic leakage 3. Wound infection 4. Pneumonia 5. Thrombosis/pulmonary embolism 6. Constipation 7. Difficulties with eating and drinking
Long-term complications	1. Internal herniation 2. Gastric ulcer 3. GERD 4. Dumping 5. Micronutrient deficiencies 6. Gallstones 7. Complaints of hypoglycemia 8. Diarrhea
Readmission	1. Readmission 2. Number of readmissions per year

*GERD* gastroesophageal reflux disease, *OSAS* obstructive sleep apnea syndrome, *COPD* chronic obstructive pulmonary disease, *QoL* quality of life, *CD* Clavien–Dindo

### Phase 2: Delphi Process

#### Expert panel

A total of 31 professionals participated in round 1. Of the 31 who completed the first round, 28 (90%) completed the second round, and 24 (77%) completed the third round. The validation round (round 4) involved 18 professionals. Table 2 presents the participants’ profession, their engagement in routine bariatric care, and their experience in bariatric care.

**Table 2:** Participant characteristics

	Round 1 *n* = 31	Round 2 *n* = 28	Round 3 *n* = 24	Validation round *n* = 18*
**Profession**				
Surgeon	12 (39)	12 (43)	11 (46)	12 (67)
Internist	4 (13)	4 (14)	2 (8)	-
Physician assistant	8 (26)	6 (21)	5 (21)	2 (11)
Other physicians	5 (16)	4 (14)	4 (17)	3 (17)
Other	2 (7)	2 (7)	2 (8)	1 (6)
**Involved in routine follow-up after MBS**				
Yes	25 (81)	22 (79)	19 (79)	13 (72)
No, only if problems arise	6 (19)	6 (21)	5 (21)	5 (28)
**Experience in follow-up after MBS**				
1–4 years	9 (29)	6 (21)	5 (21)	3 (17)
5–10 years	6 (19)	6 (21)	5 (21)	4 (22)
> 10 years	16 (52)	16 (57)	14 (58)	11 (61)

*Six experts involved in the validation round also participated in all three Delphi rounds and two experts in round one. Data are expressed as absolute frequencies and percentages (%)

*MBS* metabolic bariatric surgery

#### Round 1

The results of the Delphi rounds are shown in Supplementary Table 1. The expert panel reached consensus in round 1 on 18 items, with 16 voted as relevant and 2 as not relevant, listed by domain below:

- Short-term textbook outcome: The absence of severe postoperative complications (CD grades 3–4), re-intervention, and mortality was scored as relevant.
- Weight: Weight loss and the term to express weight loss (%TWL) were considered relevant.
- Comorbidities: (Partial) remission of hypertension, osteoarthritis, obstructive sleep apnea syndrome (OSAS), and diabetes mellitus was deemed relevant.
- QoL: QoL was scored as relevant, and consensus was reached on its scoring method (improvement score postoperative vs. preoperative).
- Psychopathology: The absence of deterioration of pre-existing psychopathology and the absence of a new addiction were considered relevant.
- Short-term complications: The absence of hemorrhage (CD 1) and constipation (CD 2) was deemed not relevant.
- Long-term complications: The absence of internal herniation (CD grades 3–5), gastric ulcer (CD grade 5), and micronutrient deficiencies (CD grades 3–5) was scored as relevant.

Based on the individual comments provided by the panelists, the following comorbidities were proposed for consideration for round 2: cardiovascular diseases, stress urinary incontinence, fertility issues, nonalcoholic steatohepatitis (NASH)/metabolic dysfunction-associated steatotic liver disease (MASLD), depression, gout, polycystic ovarian syndrome (PCOS), lipedema, migraine, intracranial hypertension, and incisional hernia. Additionally, the consideration of preoperative indication was proposed.

#### Round 2

The expert panel expressed varying opinions regarding the relevance of the short- and long-term complications according to the Clavien–Dindo (CD) classification (ranging from 1 to 5). Therefore, the research team decided to evaluate the complications as minor (CD 1 and 2) and major (CD 3 and 4) in round 2, which is a common approach to assess complications in (metabolic bariatric) surgery [22]. Moreover, the study team decided to exclude CD grade 5, as it is equivalent to mortality and thus represents an unattainable long-term outcome. In this round, consensus was reached on an additional 26 items—19 deemed relevant and 7 not relevant—listed by domain below:

- Short-term textbook outcome: Prolonged length of stay was deemed not relevant.
- Weight: The cutoff value for clinical optimal response (20% TWL), term to express recurrent weight gain (decrease %TWL), and the cutoff value of recurrent weight gain (10% TWL or 10 kg) were scored as relevant.
- Comorbidities: (Partial) remission of GERD, cardiovascular diseases, fertility issues, and NASH/MASLD was deemed relevant.
- QoL: Consensus was reached on the questionnaire to assess QoL (OBESI-Q).
- Lifestyle: Sufficient exercise and sufficient daily protein intake were scored as relevant.
- Psychopathology: The absence of binge eating disorder and anorexia nervosa was considered relevant.
- Short-term complications: Hemorrhage (CD 2), wound infection (CD 1–2), pneumonia (CD 1–2), constipation (CD 1), and difficulties with eating and drinking (CD 1–2) were scored as not relevant.
- Long-term complications: Gastric ulcer (CD 3–4), GERD (CD 3–4), dumping (CD 3–4), micronutrient deficiencies (CD 3–4), and hypoglycemia (CD 3–4) were deemed relevant, while gallstones (CD 1–2) was considered not relevant.
- Other: Preoperative indication was scored as relevant.

#### Round 3

Given the substantial number of items that failed to achieve consensus after two rounds, the study team carefully weighed the outcomes and decided not to represent most of these items in round 3, anticipating no additional insights given the continued disagreement among the expert panel (Supplementary Table 1). For some items, responses were particularly divided. For example, in the lifestyle domain, avoidance of carbonated beverages was considered relevant by 29%, not relevant by 46%, and 25% were unsure. Similarly, for the practice of separating eating and drinking, 19% deemed it relevant, 43% not relevant, and 39% were unsure.

Items related to short-term complications and comorbidities were excluded from the third round, as they were deemed less relevant to long-term outcomes and had not reached consensus in the previous two rounds. However, items such as long-term complications, recurrent weight gain, and micronutrient deficiencies were included in the third round due to their clinical relevance and the potential for achieving consensus, given their significant impact on patients’ quality of life.

In this third round, consensus was achieved on 4 items, with 1 deemed relevant and 3 not relevant:

- Micronutrient deficiencies: Persistent micronutrient deficiencies despite maximum treatment were deemed relevant, while the absence of persistent deficiencies despite oral or intramuscular suppletion was considered not relevant.
- Long-term complications: Gallstones (CD 3–4) and diarrhea (CD 1–2) were deemed not relevant.

The final COS is presented in Table 3.

**Table 3:** Final COS

**Short-term textbook outcome**
The absence of:
Prolonged length of stay
Severe postoperative complications
Re-intervention
Mortality
**Weight**
%TWL ≥ 20
**Comorbidities**
Partial or total remission of:
Hypertension
Osteoarthritis
GERD
OSAS
Diabetes mellitus
Cardiovascular diseases
Fertility issues
NASH/MASLD
**Quality of life**
Improvement of the OBESI-Q score postoperative vs. preoperative
**Micronutrient deficiencies**
The absence of persistent deficiencies despite maximum treatment
**Lifestyle**
Sufficient exercise * At least 150 min of moderate-to-vigorous intensity physical activity per week, along with muscle and bone strengthening exercises twice a week*
Sufficient protein intake/day * No consensus on the cut-off value*
**Psychopathology**
The absence of:
Deterioration of pre-existing psychopathology
Binge eating disorder
Anorexia nervosa
A new addiction
**Long-term complications**
The absence of:
Internal herniation CD 3–4
Gastric ulcers CD 3–4
GERD CD 3–4
Dumping CD 3–4
Micronutrient deficiencies CD 3–4
Complaints of hypoglycemia CD 3–4
**Other**
Achieving the preoperative indication after surgery * For example, improved diabetes mellitus regulation after surgery*

*%TWL* percentage total weight loss, *GERD* gastroesophageal reflux disease, *OSAS* obstructive sleep apnea syndrome, *NASH* non-alcoholic steatohepatitis, *MASLD* metabolic dysfunction-associated steatotic liver disease, *CD* Clavien–Dindo

### Phase 3: Validation

A total of 18 professionals participated in this validation round. Participant characteristics are presented in Table 2. Five out of nine domains were considered most relevant, as more than 75% of professionals rated them as relevant for COS, indicating that four domains were deemed less relevant. The domains “lifestyle” and “the absence of micronutrient deficiencies” received the lowest relevance scores at 56% and 61%, respectively, while “QoL” and “(partial) remission of comorbidities” had the highest scores at 94% and 89%. Nonetheless, all domains were deemed relevant by the majority of professionals. Results from voting on the final COS domains are presented in Table 4. At the end of the questionnaire, participants rated the relevance of COS for measuring and benchmarking bariatric care on a 5-point Likert scale, ranging from “not interesting at all” to very interesting.” The majority of participants (*n* = 14; 78%) rated it as interesting (score of 4), while two participants (11%) indicated a rating of 3 (neutral), and 2 others (11%) rated it as 2 (not interesting).

**Table 4:** Results validation round (round 4)

	Relevant for COS	Not relevant for COS
The absence of severe postoperative complications requiring re-interventions (CD ≥ 3)	15 (83)	3 (17)
Weight	15 (83)	3 (17)
Partial or total remission of comorbidities	16 (89)	2 (11)
QoL	17 (94)	1 (6)
The absence of micronutrient deficiencies	11 (61)	7 (39)
Lifestyle	10 (56)	8 (44)
The absence of psychopathology	13 (72)	5 (28)
The absence of long-term complications	12 (67)	6 (33)
Preoperative indication assessment	16 (89)	2 (11)

Data are expressed as absolute frequencies and percentages (%)

*COS* core outcome set, *CD* Clavien–Dindo, *QoL* quality of life

## Discussion

This study aimed to create a comprehensive COS for assessing the long-term outcome after MBS, incorporating patient-related factors through the use of the Delphi method. The final COS includes various domains: short-term TO, weight loss, remission of comorbidities, QoL, micronutrient deficiencies, lifestyle, psychopathology, long-term complications, and the preoperative indication. This COS can enhance clinical practice and scientific research by providing a standardized framework for comparing long-term results after MBS.

In 2019, a short-term TO consisting of multiple postoperative outcome parameters was created by Poelemeijer et al. [12, 23]. Most of these short-term parameters (prolonged length of stay, severe postoperative complications, re-intervention, and mortality) were considered important by our expert panel and included in our final COS. Readmission and intensive care observation were not considered important. Readmission may be considered less relevant if it is not accompanied by a re-intervention or severe postoperative complication since these items were considered important by the expert panel. Intensive care observation might be deemed less relevant as it can be employed for general monitoring and may pertain to other patient-related comorbidities rather than directly indicating postoperative complications.

Despite the development of a short-term TO by Poelemeijer et al. [12], a comprehensive long-term TO for MBS remains elusive. One of the main challenges in establishing a long-term TO for MBS lies in the multifaceted nature of its effects. Unlike many medical interventions, MBS is not merely a surgical procedure but a transformative process that significantly impacts physical health, psychological well-being, and overall QoL. These dimensions are closely interlinked and can vary widely among patients, making it challenging to develop a standardized TO framework that accurately captures the broad range of patient experiences and outcomes. Consequently, we have chosen to create a COS to address this complexity.

COSs have been established for various surgical conditions, including esophageal cancer resection surgery [24], colorectal cancer surgery [25], as well as MBS [13]. These COSs are recommended as a minimum standard for trials to ensure consistency in outcome reporting across studies, both in the short-term and long-term. In contrast, our COS specifically focuses on assessing long-term outcomes to compare or improve results after MBS. This distinct focus may have led to the extension of the COS established by Coulman et al. [13] to include lifestyle, additional obesity-related comorbidities, the absence of psychopathology, and the absence of long-term complications. Similar to Coulman et al. [13], we included QoL in our COS, but further expanded upon their findings by specifying the methodology for measuring QoL and determining how improvements in QoL should be assessed, thereby emphasizing the importance of psychological outcomes. Recently, Dijkhorst et al. [26] published a core set of patient-reported outcome measures for measuring QoL in clinical obesity care, which further underscores the significance of QoL in the context of MBS.

Additionally, physical outcomes were considered important in our COS, encompassing factors such as weight, (partial) remission of comorbidities, and lifestyle. Unexpectedly, recurrent weight gain was deemed not important by the expert panel, despite reaching a consensus on its definition (a decrease of at least 10% TWL). A possible explanation is that experts may not consider recurrent weight gain alone to be particularly important unless it is accompanied by a relapse of preoperative comorbidities or other health issues related to recurrent weight gain.

It is evident that the long-term success of MBS is determined by outcome measures that extend beyond weight loss and comorbidity reduction. The SF-Bari score, a comprehensive scoring system, represents another initiative aimed at evaluating these broader outcomes after MBS [27]. It includes metrics for weight loss, assessment of comorbidities, the Comprehensive Complication Index (CCI), and a version that incorporates QoL evaluations [27]. The scores range from suboptimal to excellent, providing a valuable tool for evaluating MBS outcomes [27]. The results of our study add valuable information to the promising SF-Bari score. While the SF-Bari score uses the CCI based on the Clavien–Dindo classification to evaluate complications, our study goes further by exploring which complications healthcare professionals consider most important. This additional perspective enhances the utility of the SF-Bari score in assessing MBS outcomes.

### Strengths and Limitations

The strengths of this study include the development of a COS to evaluate long-term clinical outcomes after MBS. Additionally, a proper study design was used, namely a Delphi consensus. Furthermore, we not only identified which outcomes should be measured but also examined how most of these outcomes should be assessed.

Our study has some limitations that need to be mentioned: first, the sample size of participants was small. However, there are no established guidelines for determining a statistically representative sample in Delphi surveys [21]. Second, the distribution between surgeons and internists could be improved, as more surgeons were included, and only surgeons were involved in the validation process. However, we also included PAs and other physicians, who could provide different perspectives on the COS due to their diverse medical backgrounds. Third, an important group was overlooked, namely the patients. In the future, it will be essential to expand the COS to include all stakeholder groups, particularly patients. Fourth, we did not provide definitions for “micronutrient deficiencies” or “complaints of hypoglycemia,” which may have led to varying interpretations by the expert panel. However, standardized laboratory tests are recommended after MBS according to national Dutch guidelines, ensuring consistency across the Netherlands [28]. This suggests that most professionals likely view these tests similarly to our reference to “micronutrient deficiencies.” Finally, this study included only Dutch physicians. Future studies could conduct an international Delphi consensus to determine if the Dutch COS is generalizable to other countries.

## Conclusion

In this study, a comprehensive COS was developed, which can enhance clinical practice and scientific research by providing a standardized framework for comparing long-term results after MBS. The final COS includes key domains such as short-term TO, weight loss, remission of comorbidities, QoL, micronutrient deficiencies, lifestyle, psychopathology, long-term complications, and preoperative indication. These selected domains represent outcomes that are most important to healthcare professionals and should be considered the minimum standard for assessing outcomes in clinical practice. Follow-up after MBS should focus not only on weight loss and the remission of comorbidities but also on these additional important domains.

## Supplementary Information

Below is the link to the electronic supplementary material.

Supplementary file1 (DOCX 77 KB)

## Data Availability

Data is provided within the manuscript or supplementary information files.
