# Supplementary material for: DELTO Study: Delphi Consensus on Long-Term Textbook Outcome After Metabolic Bariatric Surgery
Source: Obes Surg. 2025 Jan 18;35(2):535–43. doi: 10.1007/s11695-024-07587-6 (PMC11836189; doi:10.1007/s11695-024-07587-6)
Supplement: Supplementary file 1 — Supplementary file1 (DOCX 77 KB) [file 11695_2024_7587_MOESM1_ESM.docx]

| **Table 1. Consensus during three Delphi rounds** | | | | | | | | | |
| --- | --- | --- | --- | --- | --- | --- | --- | --- | --- |
|  | **Round 1**  **n = 31** | | | **Round 2**  **n = 28** | | | **Round 3**  **n = 24** | | |
| Outcome | Relevant | Not relevant | Unsure | Relevant | Not relevant | Unsure | Relevant | Not relevant | Unsure |
| Short-term textbook outcome | | | | | | | | | |
| Prolonged length of stay | 10 (32) | 16 (52) | 5 (16) | 6 (21) | **21 (75)** | 1 (4) | - | - | - |
| Readmission | 16 (52) | 10 (32) | 5 (16) | 9 (32) | 17 (61) | 2 (7) | -* | -* | -* |
| Severe postoperative complications | **26 (84)** | 1 (3) | 4 (13) | - | - | - | - | - | - |
| Re-intervention | **25 (81)** | 4 (13) | 2 (7) | - | - | - | - | - | - |
| Intensive care observation | 17 (55) | 6 (19) | 8 (26) | 16 (57) | 10 (36) | 2 (7) | -* | -* | -* |
| Mortality | **26 (84)** | 1 (3) | 4 (13) | - | - | - | - | - | - |
| Weight | | | | | | | | | |
| Weight loss | **100 (100)** | 0 (0) | 0 (0) | - | - | - | - | - | - |
| Term weight loss  - %TWL  - %EWL  - Other | **24 (77)**  6 (19)  1 (3) | -  -  - | -  -  - | -  -  - | -  -  - | -  -  - | -  -  - | -  -  - | -  -  - |
| Cutoff value clinical optimal response  In %TWL  - 10  - 15  - 20  - 23  - 25  - 60  In %EWL  - 50  - 60  - 66  - 70 | 1 (3)  3 (10)  10 (32)  1 (3)  9 (29)  1 (3)  3 (50)  1 (17)  1 (17)  1 (17) | -  -  -  -  -  -  -  -  -  - | -  -  -  -  -  -  -  -  -  - | -  -  **22 (79)**  -  6 (21)  -  -  -  -  - | -  -  -  -  -  -  -  -  -  - | -  -  -  -  -  -  -  -  -  - | -  -  -  -  -  -  -  -  -  - | -  -  -  -  -  -  -  -  -  - | -  -  -  -  -  -  -  -  -  - |
| Recurrent weight gain | 21 (68) | 6 (19) | 4 (13) | 14 (50) | 13 (46) | 1 (4) | 15 (63) | 6 (25) | 3 (13) |
| Term to express recurrent weight gain  - Decrease %TWL  - Decrease %EWL  - Weight gain in kg from nadir weight  - Other | 16 (52)  2 (7)  11 (36)  2 (7) | -  -  -  - | -  -  -  - | **22 (79)**  -  6 (21)  - | -  -  -  - | -  -  -  - | -  -  -  - | -  -  -  - | -  -  -  - |
| Cutoff value recurrent weight gain  In %TWL  - 5  - 10  - 15  - 20  - 30  In %EWL  - 30  - 50  In kg  - 5  - 10  - 15  - 19  - 25  - 30 | 5 (31)  7 (44)  1 (6)  2 (13)  1 (6)    1 (50)  1 (50)    2 (18)  4 (36)  2 (18)  1 (9)  1 (9)  1 (9) | -  -  -  -  -  -  -  -  -  -  -  -  - | -  -  -  -  -  -  -  -  -  -  -  -  - | 6 (21)  **22 (79)**  -  -  -  -  -  0 (0)  **21 (75)**  7 (25)  -  -  - | -  -  -  -  -  -  -  -  -  -  -  -  - | -  -  -  -  -  -  -  -  -  -  -  -  - | -  -  -  -  -  -  -  -  -  -  -  -  - | -  -  -  -  -  -  -  -  -  -  -  -  - | -  -  -  -  -  -  -  -  -  -  -  -  - |
| Comorbidities | | | | | | | | | |
| Hypertension | **29 (94)** | 1 (3) | 1 (3) | - | - | - | - | - | - |
| Dyslipidemia | 23 (74) | 1 (3) | 7 (23) | 17 (61) | 2 (7) | 9 (32) | -* | -* | -* |
| Osteoarthritis | **24 (77)** | 2 (7) | 5 (16) | - | - | - | - | - | - |
| GERD | 23 (74) | 1 (3) | 7 (23) | **25 (89)** | 0 (0) | 3 (11) | - | - | - |
| OSAS | **31 (100)** | 0 (0) | 0 (0) | - | - | - | - | - | - |
| Diabetes mellitus | **31 (100)** | 0 (0) | 0 (0) | - | - | - | - | - | - |
| Asthma/ COPD | 20 (65) | 1 (3) | 10 (32) | 18 (64) | 3 (11) | 7 (25) | -* | -* | -* |
| Cardiovascular diseases | - | - | - | **22 (79)** | 3 (11) | 3 (11) | - | - | - |
| Stress urinary incontinence | - | - | - | 9 (32) | 9 (32) | 10 (36) | -* | -* | -* |
| Fertility issues | - | - | - | **21 (75)** | 3 (11) | 4 (14) | - | - | - |
| NASH/MASLD | - | - | - | **27 (97)** | 0 (0) | 1 (4) | - | - | - |
| Depression | - | - | - | 14 (50) | 4 (14) | 10 (36) | -* | -* | -* |
| Gout | - | - | - | 7 (25) | 6 (21) | 15 (54) | -* | -* | -* |
| PCOS | - | - | - | 20 (71) | 3 (11) | 5 (18) | -* | -* | -* |
| Lipedema | - | - | - | 8 (29) | 7 (25) | 13 (46) | -* | -* | -* |
| Migraine | - | - | - | 7 (25) | 5 (18) | 16 (57) | -* | -* | -* |
| Intracranial hypertension | - | - | - | 17 (61) | 2 (7) | 9 (32) | -* | -* | -* |
| Incisional hernia | - | - | - | 15 (54) | 4 (14) | 9 (32) | -* | -* | -* |
| Quality of life | | | | | | | | | |
| QoL | **31 (100)** | 0 (0) | 0 (0) | - | - | - | - | - | - |
| Questionnaire to assess QoL  - IWQOL-Lite  - BAROS  - OBESI-Q  - PROM  - QALY  - SF-36  - PROM + OBESI-Q  - Unsure  - Other | 3 (10)  1 (3)  15 (48)  1 (3)  1 (3)  2 (6)  3 (10)  -  3 (10) | -  -  -  -  -  -  -  -  - | -  -  -  -  -  -  -  2 (6)  - | 1 (4)  -  **27 (96)**  -  -  0 (0)  -  -  - | -  -  -  -  -  -  -  -  - | -  -  -  -  -  -  -  -  - | -  -  -  -  -  -  -  -  - | -  -  -  -  -  -  -  -  - | -  -  -  -  -  -  -  -  - |
| Method of scoring QoL - Improvement score postoperative vs preoperative  - Above desired cutoff value  - Other | **29 (94)**  1 (3)  1 (3) | -  -  - | -  -  - | -  -  - | -  -  - | -  -  - | -  -  - | -  -  - | -  -  - |
| Micronutrient deficiencies | | | | | | | | | |
| Micronutrient deficiencies | 22 (71) | 3 (10) | 6 (19) | 19 (68) | 7 (25) | 2 (7) | 15 (63) | 6 (25) | 3 (13) |
| Severity of the deficiency  - No deficiencies  - Oral suppletion  - Oral or i.m. suppletion  - Oral, i.m. or i.v. suppletion  - Persistent deficiencies despite maximum treatment | 1 (3)  5 (16)  9 (29)  7 (23)  9 (29) | -  -  -  -  - | -  -  -  -  - | -  -  14 (50)  9 (32)  5 (18) | -  -  -  -  - | -  -  -  -  - | -  -  0 (0)  4 (17)  **19 (79)** | -  -  **24 (100)**  16 (67)  3 (13) | -  -  0 (0)  4 (17)  2 (8) |
| Lifestyle | | | | | | | | | |
| Sufficient exercise | 23 (74) | 2 (7) | 6 (19) | **25 (89)** | 1 (4) | 2 (7) | - | - | - |
| Sufficient mealtimes/day | 15 (48) | 4 (13) | 12 (39) | 16 (57) | 6 (21) | 6 (21) | -* | -* | -* |
| Sufficient protein intake/day | 23 (74) | 2 (7) | 6 (19) | **25 (89)** | 1 (4) | 2 (7) | - | - | - |
| Cutoff value sufficient protein intake/day  - < 60 gr/day  - 60-90 gr/day  - > 90 gr/day  - 0.8 gr protein per kg of body weight per day  - > 0.8 gr protein per kg of body weight per day  - Other | 0 (0)  11 (36)  1 (3)  9 (29)  8 (26)  2 (6) | -  -  -  -  -  - | -  -  -  -  -  - | -  11 (39)  -  10 (36)  7 (25)  - | -  -  -  -  -  - | -  -  -  -  -  - | -  -*  -  -*  -*  - | -  -*  -  -*  -*  - | -  -*  -  -*  -*  - |
| Separate eating and drinking | 9 (29) | 11 (36) | 11 (36) | 5 (18) | 12 (43) | 11 (39) | -* | -* | -* |
| Avoidance of carbonated beverages | 10 (32) | 13 (26) | 8 (42) | 8 (29) | 13 (46) | 7 (25) | -* | -* | -* |
| Psychopathology | | | | | | | | | |
| Deterioration of pre-existing psychopathology | **27 (87)** | 1 (3) | 3 (10) | - | - | - | - | - | - |
| Binge eating disorder | 22 (71) | 5 (16) | 4 (13) | **24 (86)** | 3 (11) | 1 (4) | - | - | - |
| Severity binge eating disorder  - Vomiting  - Recurrent weight gain  - Dumping  - Psychological complaints  - Other | 4 (13)  8 (36)  2 (7)  4 (13)  13 (42) | -  -  -  -  - | -  -  -  -  - | 10 (36)  14 (50)  -  4 (14)  - | -  -  -  -  - | -  -  -  -  - | -*  -*  -  -*  - | -*  -*  -  -*  - | -*  -*  -  -*  - |
| Anorexia nervosa | 23 (74) | 2 (7) | 6 (19) | **26 (93)** | 1 (4) | 1 (4) | - | - | - |
| Severity anorexia nervosa  - Hypoglycemia  - BMI < 18.5  - Vitamin and mineral deficiencies  - Need for enteral or parenteral feeding  - Psychiatric treatment  - Other | 2 (7)  9 (29)  1 (3)  4 (13)  3 (10)  12 (39) | -  -  -  -  -  - | -  -  -  -  -  - | -  16 (57)  -  7 (25)  5 (18)  - | -  -  -  -  -  - | -  -  -  -  -  - | -  -*  -  -*  -*  - | -  -*  -  -*  -*  - | -  -*  -  -*  -*  - |
| Problems with depression / depressive symptoms | 20 (65) | 4 (13) | 7 (23) | 16 (57) | 6 (21) | 6 (21) | -* | -* | -* |
| Development of a new addiction | **26 (84)** | 2 (7) | 3 (10) | - | - | - | - | - | - |
| Short-term complications ◦ | | | | | | | | | |
| Hemorrhage |  |  |  | 6 (21) | 18 (64) | 4 (14) | - | - | - |
| - CD1 | 3 (10) | **24 (77)** | 4 (13) | 2 (7) | **23 (82)** | 3 (11) | - | - | - |
| - CD2 | 8 (28) | 19 (66) | 2 (7) |  |  |  |  |  |  |
| - CD3 | 12 (41) | 10 (35)\| | 7 (24) | 14 (50) | 12 (43) | 2 (7) | -* | -* | -* |
| - CD4 | 15 (52) | 8 (28) | 6 (21) |  |  |  |  |  |  |
| - CD5 | 20 (6) | 8 (28) | 1 (3) | - | - | - | - | - | - |
| Anastomotic leakage |  |  |  | 12 (43) | 11 (39) | 5 (18) | - | - | - |
| - CD1 | 5 (16) | 23 (74) | 3 (10) | 6 (22) | 20 (71) | 2 (7) | -* | -* | -* |
| - CD2 | 6 (21) | 18 (62) | 5 (17) |  |  |  |  |  |  |
| - CD3 | 15 (52) | 5 (17) | 9 (31) | 19 (68) | 8 (29) | 1 (4) | -* | -* | -* |
| - CD4 | 21 (72) | 3 (10) | 5 (17) |  |  |  |  |  |  |
| - CD5 | 21 (72) | 5 (17) | 3 (10) | - | - | - | - | - | - |
| Wound infection |  |  |  | 3 (11) | **23 (82)** | 2 (7) | - | - | - |
| - CD1 | 5 (16) | 22 (71) | 4 (13) | 4 (14) | **23 (82)** | 1 (4) | - | - | - |
| - CD2 | 7 (24) | 20 (69) | 2 (7) |  |  |  |  |  |  |
| - CD3 | 6 (21) | 15 (52) | 8 (28) | 9 (32) | 14 (50) | 5 (18) | -* | -* | -* |
| - CD4 | 13 (45) | 10 (34) | 6 (21) |  |  |  |  |  |  |
| - CD5 | 17 (59) | 9 (31) | 3 (10) | - | - | - | - | - | - |
| Pneumonia |  |  |  | 4 (14) | 19 (68) | 5 (18) | - | - | - |
| - CD1 | 5 (16) | 21 (68) | 5 (16) | 1 (4) | **22 (79)** | 5 (18) | - | - | - |
| - CD2 | 8 (28) | 16 (55) | 5 (17) |  |  |  |  |  |  |
| - CD3 | 7 (24) | 14 (48) | 8 (28) | 11 (39) | 11 (39) | 6 (21) | -* | -* | -* |
| - CD4 | 13 (45) | 8 (28) | 8 (28) |  |  |  |  |  |  |
| - CD5 | 19 (66) | 8 (28) | 2 (7) | - | - | - | - | - | - |
| Thrombosis / pulmonary embolism |  |  |  | 4 (14) | 16 (57) | 5 (18) | - | - | - |
| - CD1 | 5 (16) | 22 (71) | 4 (13) | 1 (4) | 19 (68) | 5 (18) | -* | -* | -* |
| - CD2 | 11 (38) | 12 (41) | 6 (21) |  |  |  |  |  |  |
| - CD3 | 13 (45) | 12 (41) | 4 (14) | 11 (39) | 11 (39) | 6 (21) | -* | -* | -* |
| - CD4 | 18 (62) | 6 (21) | 5 (17) |  |  |  |  |  |  |
| - CD5 | 19 (66) | 8 (28) | 2 (7) | - | - | - | - | - | - |
| Constipation |  |  |  | 3 (11) | 21 (75) | 4 (14) | - | - | - |
| - CD1 | 7 (23) | 21 (68) | 3 (10) | 2 (7) | **23 (82)** | 3 (11) | - | - | - |
| - CD2 | 5 (17) | **22 (76)** | 2 (7) |  |  |  |  |  |  |
| - CD3 | 5 (17) | 18 (62) | 6 (21) | 5 (18) | 16 (57) | 7 (25) | -* | -* | -* |
| - CD4 | 11 (38) | 12 (41) | 6 (21) |  |  |  |  |  |  |
| - CD5 | 18 (62) | 10 (35) | 1 (3) | - | - | - | - | - | - |
| Difficulties with eating and drinking |  |  |  | 2 (7) | 19 (68) | 7 (25) | - | - | - |
| - CD1 | 13 (41) | 16 (52) | 2 (7) | 2 (7) | **21 (75)** | 5 (18) | - | - | - |
| - CD2 | 9 (31) | 16 (55) | 4 (14) |  |  |  |  |  |  |
| - CD3 | 10 (35) | 13 (45) | 6 (21) | 14 (50) | 8 (29) | 6 (21) | -* | -* | -* |
| - CD4 | 16 (55) | 9 (31) | 4 (14) |  |  |  |  |  |  |
| - CD5 | 18 (62) | 10 (35) | 1 (3) | - | - | - | - | - | - |
| Long-term complications ◦ | | | | | | | | | |
| Internal herniation |  |  |  | 18 (64) | 9 (32) | 1 (4) |  |  |  |
| - CD1 | 9 (29) | 16 (52) | 6 (19) | 14 (50) | 12 (43) | 2 (7) | 10 (42) | 12 (50) | 2 (8) |
| - CD2 | 13 (45) | 11 (38) | 5 (17) |  |  |  |  |  |  |
| - CD3 | **22 (76)** | 4 (14) | 3 (10) | **22 (79)** | 4 (14) | 2 (7) | - | - | - |
| - CD4 | **24 (83)** | 1 (3) | 4 (14) |  |  |  |  |  |  |
| - CD5 | **24 (83)** | 3 (10) | 2 (7) | - | - | - | - | - | - |
| Gastric ulcer |  |  |  | 15 (54) | 7 (25) | 6 (21) | - | - | - |
| - CD1 | 10 (32) | 14 (45) | 7 (23) | 18 (64) | 8 (29) | 2 (7) | 16 (67) | 7 (29) | 1 (4) |
| - CD2 | 17 (59) | 7 (24) | 5 (17) |  |  |  |  |  |  |
| - CD3 | 20 (69) | 4 (14) | 5 (17) | **23 (82)** | 2 (7) | 3 (11) | - | - | - |
| - CD4 | 21 (72) | 1 (3) | 7 (24) |  |  |  |  |  |  |
| - CD5 | **22 (76)** | 6 (21) | 1 (3) | - | - | - | - | - | - |
| GERD |  |  |  | 17 (61) | 6 (22) | 5 (18) | - | - | - |
| - CD1 | 13 (42) | 10 (32) | 8 (26) | 17 (61) | 6 (22) | 5 (18) | 15 (63) | 8 (33) | 1 (4) |
| - CD2 | 18 (62) | 7 (24) | 4 (14) |  |  |  |  |  |  |
| - CD3 | 18 (62) | 5 (17) | 6 (21) | **22 (79)** | 2 (7) | 4 (14) | - | - | - |
| - CD4 | 17 (59) | 7 (24) | 5 (17) |  |  |  |  |  |  |
| - CD5 | 18 (62) | 10 (35) | 1 (3) | - | - | - | - | - | - |
| Dumping |  |  |  | 15 (54) | 5 (18) | 8 (29) | - | - | - |
| - CD1 | 12 (39) | 8 (26) | 11 (36) | 13 (46) | 8 (29) | 7 (25) | 8 (33) | 12 (50) | 4 (17) |
| - CD2 | 17 (59) | 7 (24) | 5 (17) |  |  |  |  |  |  |
| - CD3 | 17 (61) | 7 (24) | 4 (14) | **24 (86)** | 2 (7) | 2 (7) | - | - | - |
| - CD4 | 19 (68) | 5 (17) | 4 (14) |  |  |  |  |  |  |
| - CD5 | 19 (68) | 8 (28) | 1 (3) | - | - | - | - | - | - |
| Malnutrition |  |  |  | **22 (79)** | 4 (14) | 2 (7) | - | - | - |
| - CD1 | 13 (42) | 11 (36) | 7 (23) | **23 (82)** | 3 (11) | 2 (7) | - | - | - |
| - CD2 | 20 (69) | 3 (10) | 6 (21) |  |  |  |  |  |  |
| - CD3 | **24 (83)** | 1 (3) | 4 (14) | **26 (93)** | 1 (4) | 1 (4) | - | - | - |
| - CD4 | **23 (79)** | 3 (10) | 3 (10) |  |  |  |  |  |  |
| - CD5 | **22 (76)** | 5 (17) | 2 (7) | - | - | - | - | - | - |
| Gallstones |  |  |  | 5 (18) | 17 (61) | 6 (21) | - | - | - |
| - CD1 | 8 (26) | 16 (52) | 7 (23) | 3 (11) | **22 (79)** | 3 (11) | - | - | - |
| - CD2 | 13 (45) | 10 (35) | 6 (21) |  |  |  |  |  |  |
| - CD3 | 12 (41) | 9 (31) | 28) | 9 (32) | 14 (50) | 5 (18) | 2 (8) | **21 (88)** | 1 (4) |
| - CD4 | 11 (38) | 9 (31) | 9 (31) |  |  |  |  |  |  |
| - CD5 | 15 (52) | 11 (38) | 3 (10) | - | - | - | - | - | - |
| Complaints of hypoglycemia |  |  |  | 19 (68) | 5 (18) | 4 (14) | - | - | - |
| - CD1 | 12 (39) | 10 (32) | 9 (29) | 14 (50) | 6 (21) | 8 (29) | 16 (67) | 7 (29) | 1 (4) |
| - CD2 | 15 (52) | 4 (14) | 10 (35) |  |  |  |  |  |  |
| - CD3 | 20 (69) | 5 (17) | 4 (14) | **21 (75)** | 3 (11) | 4 (14) | - | - | - |
| - CD4 | 21 (72) | 5 (17) | 3 (10) |  |  |  |  |  |  |
| - CD5 | 21 (72) | 7 (24) | 1 (3) | - | - | - | - | - | - |
| Diarrhea |  |  |  | 9 (32) | 10 (36) | 9 (32) | - | - | - |
| - CD1 | 10 (32) | 11 (36) | 10 (32) | 9 (32) | 12 (43) | 7 (25) | 1 (4) | **19 (79)** | 4 (17) |
| - CD2 | 16 (55) | 8 (28) | 5 (17) |  |  |  |  |  |  |
| - CD3 | 15 (52) | 8 (28) | 6 (21) | 16 (57) | 7 (25) | 5 (18) | 14 (58) | 5 (21) | 5 (21) |
| - CD4 | 19 (66) | 5 (17) | 5 (17) |  |  |  |  |  |  |
| - CD5 | 20 (69) | 6 (21) | 3 (10) | - | - | - | - | - | - |
| Hypoalbuminemia | - | - | - | 6 (21) | 8 (29) | 14 (50) | - | - | - |
| Osteoporosis | - | - | - | 12 (43) | 11 (39) | 5 (18) | - | - | - |
| Ileus | - | - | - | 17 (61) | 5 (18) | 6 (21) | - | - | - |
| Readmission | | | | | | | | | |
| Readmission | 14 (45) | 12 (39) | 5 (16) | 6 (21) | 19 (68) | 3 (11) | -* | -* | -* |
| Number of readmissions per year  - 1  - 2  - 3  - Other | 11 (36)  10 (32)  2 (7)  8 (26) | -  -  -  - | -  -  -  - | 15 (54)  13 (46)  -  - | -  -  -  - | -  -  -  - | -*  -*  -  - | -*  -*  -  - | -*  -*  -  - |
| Other | | | | | | | | | |
| Preoperative indication | - | - | - | **25 (89)** | 1 (4) | 2 (7) | - | - | - |
| Data expressed as absolute frequencies and percentages (%).  ◦ In the first round, the number of respondents for CD1 was 31, while for CD2-5, it was 29.  * Item was not reconsidered due to a lack of consensus in previous round(s).  %TWL = percentage Total Weight Loss; %EWL = percentage Excess Weight Loss; GERD = Gastroesophageal Reflux Disease; OSAS = Obstructive Sleep Apnea Syndrome; COPD = Chronic obstructive pulmonary disease; NASH = Non-Alcoholic Steatohepatitis; MASLD = Metabolic Dysfunction-Associated Steatotic Liver Disease; QoL = Quality of Life; BMI = Body Mass Index, kg/m^2^; CD = Clavien-Dindo. | | | | | | | | | |
